# LUCID: An Integrative Approach for Target Discovery and dsRNA Design in Plant Fungal Pathogens

**DOI:** 10.1111/pbi.70526

**Published:** 2026-02-12

**Authors:** Lucía Jiménez‐Castro, Alba López‐Laguna, Dolores Férnandez‐Ortuño, Alejandro Pérez‐García, Álvaro Polonio

**Affiliations:** ^1^ Departamento de Microbiología, Facultad de Ciencias Universidad de Málaga Málaga Spain; ^2^ Instituto de Hortofruticultura Subtropical y Mediterránea ‘La Mayora’ Universidad de Málaga, Consejo Superior de Investigaciones Científicas (IHSM−UMA−CSIC) Málaga Spain

**Keywords:** biofungicides, *botrytis cinerea*, comparative genomics, dsRNA, phytopathogenic fungi, RNAi, transcriptomics

## Abstract

Phytopathogenic fungi pose an escalating threat to global food security and ecosystem stability, as resistance and environmental concerns diminish the effectiveness of conventional fungicides. Double‐stranded RNA (dsRNA)‐based fungicides offer a species‐specific, eco‐friendly alternative. We introduce LUCID (Locating Uncovered, conserved, and Indispensable for pathogenicity Determinants), a computational pipeline that accelerates the development of RNAi‐based biofungicides by integrating target identification with dsRNA design and off‐target prediction. LUCID employs a dual‐branch strategy to identify both Conserved Essential Proteins (CEPs) and Conserved Non‐Annotated Proteins (CNAPs), leveraging transcriptomic data and comparative genomics across diverse fungal species. Validation in *Botrytis cinerea* demonstrated high efficacy, with 67% of proposed targets successfully silenced and an average silencing efficiency of 96%. Additionally, coupling LUCID with advanced protein language models (PLMs) revealed a novel pathogenicity determinant in *B. cinerea* : a putative mediator complex protein. LUCID offers a scalable, species‐agnostic framework for designing sustainable fungicides, enabling rapid, targeted control of fungal diseases with minimal ecological impact.

## Graphical abstract

LUCID: A computational pipeline for RNAi‐based biofungicide design.

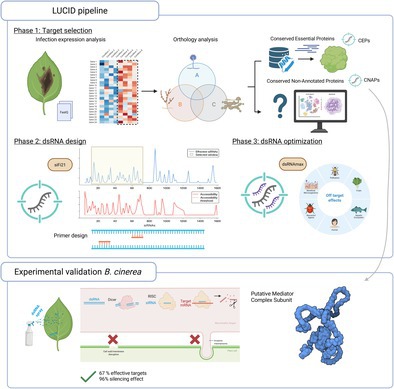

## 1 Introduction

Phytopathogenic fungi represent a significant threat to global food security and human livelihoods (Fisher et al. 2012). These pathogens can cause severe crop losses and substantial economic disruption. Recent models forecast a marked rise in both the diversity and invasion potential of these fungi across forests and agricultural lands in the coming years. The implications are alarming: such fungal diseases could compromise food availability for large segments of the global population and diminish forest‐based CO_2_ absorption by an estimated 230 to 580 megatons annually (Li et al. 2023).

Traditional management strategies, such as chemical fungicide application, have proven insufficient in tackling the challenges posed by many phytopathogenic fungi. The widespread use of broad‐spectrum fungicides has driven the emergence of resistant fungal strains, complicating disease control efforts. Fungicide resistance, an inheritable trait resulting in reduced fungal sensitivity, often arises from the overuse and misuse of chemical treatments, which exert selective pressure on fungal populations, enabling resistant strains to survive and proliferate. Consequently, many once‐effective fungicides have lost their efficacy, underscoring the urgent need for alternative, sustainable strategies to combat fungal phytopathogens (Jiang et al. 2025).

In this scenario, there is a need to develop new antifungal agents that function through innovative mechanisms and minimise environmental harm. Promoting sustainable agricultural practices is essential for enhancing environmental quality and optimising resource use (Ansari et al. 2012). Regulatory bodies in both the European Union and the United States have already prohibited several chemical pesticides due to their detrimental effects, and many conventional products have been phased out over time due to rising concerns about their ecological and health risks (Damalas and Eleftherohorinos 2011; Chattopadhyay et al. 2017).

Emerging technologies offer promising solutions to the growing challenges of fungal disease management in agriculture. Among these, double‐stranded RNAs (dsRNAs) have gained attention as a targeted and environmentally sustainable alternative to conventional fungicides (Wang et al. 2016; Padilla‐Roji et al. 2023). Unlike broad‐spectrum chemical treatments that can harm non‐target organisms, dsRNAs function through RNA interference (RNAi), a biological process in which specific dsRNA molecules silence genes critical to fungal growth and pathogenicity by matching their genetic sequences (Padilla‐Roji et al. 2023). This precision allows for minimal disruption to the surrounding ecosystem (Neumeier and Meister 2021). Furthermore, dsRNAs are biodegradable, breaking down rapidly without leaving harmful residues in the environment (Parker et al. 2019). A key advantage of this approach is its potential to limit resistance development. Because dsRNAs can be engineered to target multiple essential genes and suppress transcript expression rather than bind to protein active sites, fungal pathogens face greater difficulty adapting to these treatments (Sundaresha et al. 2022). Altogether, dsRNA‐based strategies present a more resilient and ecologically responsible path forward for controlling fungal diseases in crops.

Recent advances have demonstrated the strong potential of dsRNA‐based strategies in managing fungal diseases (Koch and Kogel 2014; Qiao et al. 2021). Notable examples include multiple fungal species like *Sclerotinia sclerotiorum*, *Rhizoctonia solani*, *Aspergillus niger*, *Botrytis cinerea*, and *Fusarium* species, with each pathogen showing efficient dsRNA uptake through clathrin‐mediated endocytosis (Duanis‐Assaf et al. 2022; Bocos‐Asenjo et al. 2025; Chen, Imran, et al. 2025). The effectiveness of these approaches relies heavily on precise design and delivery, with critical factors including the selection of suitable gene targets and the identification of optimal regions for siRNA production (Ray et al. 2022; Mosquera et al. 2025). Commercialization of SIGS is underway, led by GreenLight Biosciences (Pallis et al. 2023), with Calantha (the first EPA‐approved foliar RNA bioinsecticide) and expanding efforts to combat fungal diseases in crops like strawberries and grapes. With large‐scale production capabilities and growing industry investment, SIGS is transitioning from research to practical application.

However, unlocking the full potential of dsRNA‐based fungicides requires efficient computational tools for target selection. Current approaches rely on species‐specific bioinformatics pipelines and labour‐intensive screening, lacking standardised solutions for broad application across diverse phytopathogens (McLoughlin et al. 2018; Ruiz‐Jiménez et al. 2021). To address this gap, we developed LUCID, a three‐phase computational framework that integrates RNA‐seq data and comparative genomics for target identification, followed by automated dsRNA design, primer generation and off‐target prediction. We applied LUCID to *B. cinerea* , a widespread fungal pathogen responsible for grey mould in numerous crops and known for its resistance to multiple fungicides, which complicates effective control (López‐Laguna et al. 2025). LUCID allowed the identification of targets that significantly reduced disease symptoms, including both essential and novel pathogenicity‐related proteins, establishing LUCID as a robust and scalable tool for developing RNAi‐based biofungicides across a wide range of fungal pathogens.

## 2 Results

### 2.1 LUCID Pipeline Allows Automated Target Selection and dsRNA Design

**FIGURE 1:**
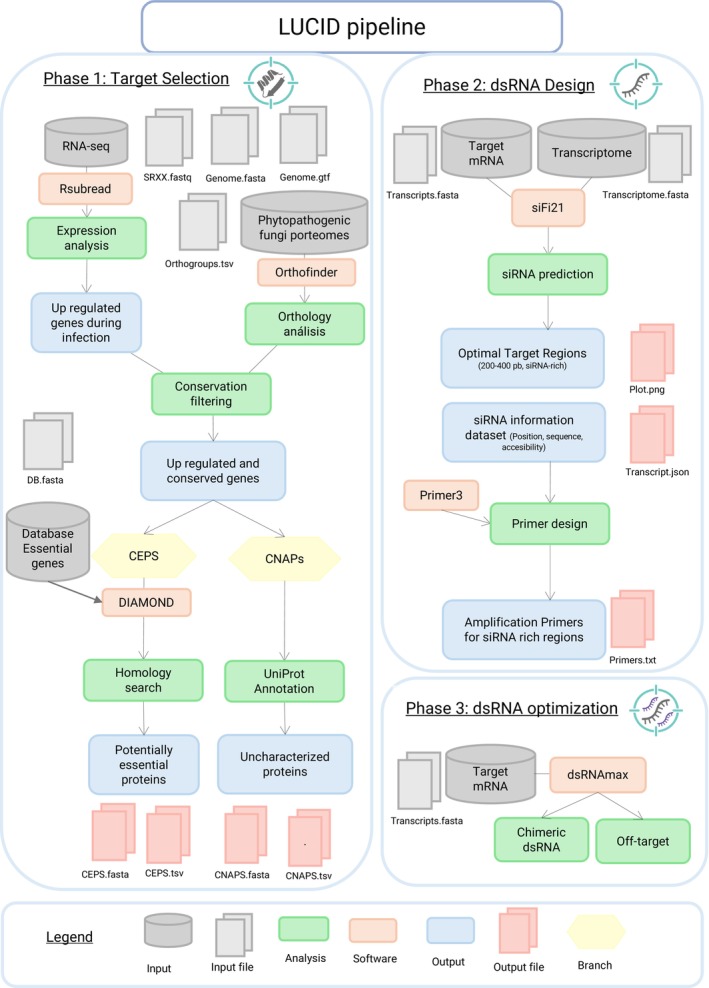
Overview of the LUCID bioinformatic pipeline. The LUCID workflow comprises two interconnected phases. Phase 1: Target Selection (left panel), Phase 2: DsRNA Design (upper right panel), and Phase 3: DsRNA Optimization (lower right panel). In Phase 1, RNA‐seq data and proteomes from phytopathogenic fungi are integrated to identify candidate RNAi targets, resulting in two output categories: Conserved Essential Proteins (CEPs) and Conserved Non‐Annotated Proteins (CNAPs). Phase 2 processes these selected transcripts to design effective dsRNA molecules, using a customised version of siFi21 to pinpoint high‐efficiency silencing regions, visualise siRNA distribution, and generate primers via Primer3. Phase 3 employs dsRNAmax to design chimeric dsRNAs and assess off‐target effects against non‐target organisms. This streamlined pipeline facilitates the rapid identification and design of RNA‐based fungicides targeting fungal pathogens.

To streamline the development of RNAi‐based biofungicides, we created LUCID, a fully automated bioinformatics pipeline that integrates target selection with dsRNA design. LUCID operates in three phases (Figure 1). Phase 1: Target Selection. This phase combines RNA‐seq data, comparative genomics, and curated fungal databases to identify candidate targets in phytopathogenic fungi. Highly expressed genes during plant infection are detected via differential expression analysis or TPM (transcripts per million) values for obligate biotrophs. These transcripts are then filtered for conservation across agriculturally relevant fungal proteomes and cross‐referenced with databases of essential and infection‐related proteins to define a set of Conserved Essential Proteins (CEPs). In parallel, the pipeline identifies Conserved Non‐Annotated Proteins (CNAPs), novel, upregulated proteins conserved across pathogens but lacking functional annotation. Phase 2: dsRNA Design. Selected transcripts are processed to identify optimal silencing regions, generate dsRNA molecules, and design primers for amplification. This integrated workflow facilitates rapid and scalable development of dsRNA constructs tailored to a wide range of phytopathogenic fungi. Phase 3: Off‐Target Prediction. Designed dsRNAs are evaluated for potential cross‐reactivity against user‐provided non‐target transcriptomes, and validated targets can be combined into multi‐target chimeric dsRNAs.

### 2.2 Differential Expression and Conservation Analysis Reveal Core Fungal Pathogenicity Arsenal

**FIGURE 2:**
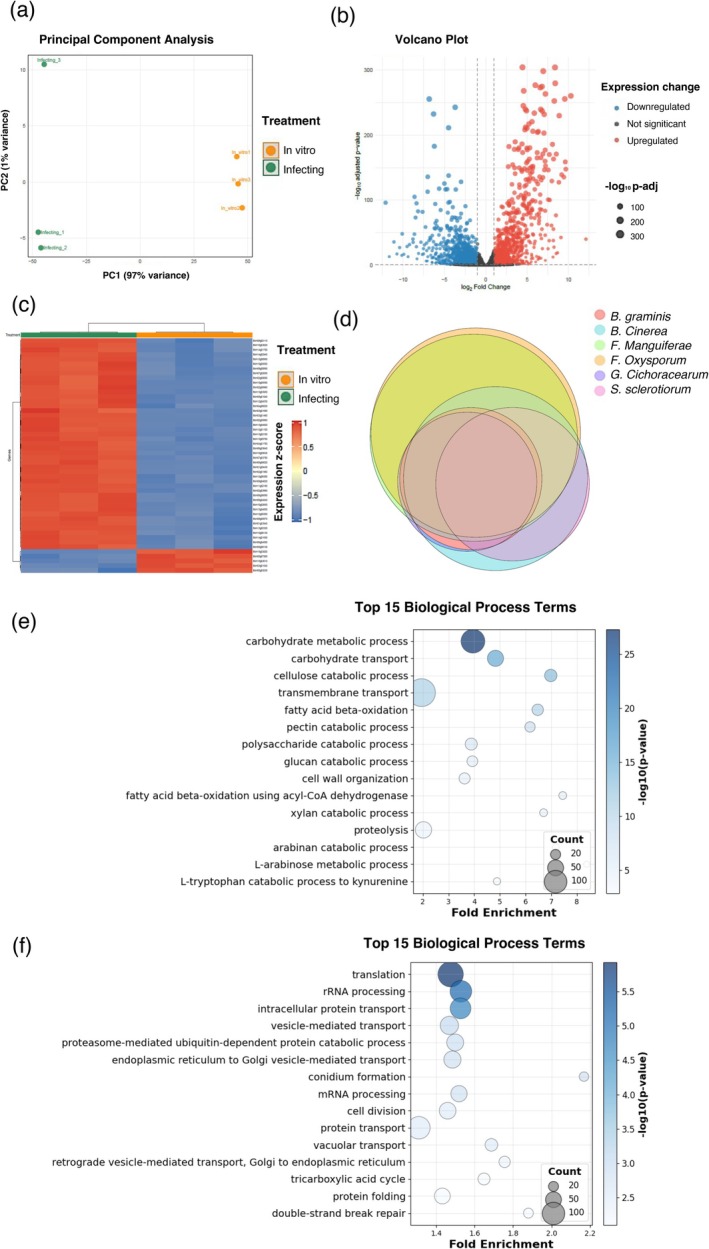
Transcriptomic and comparative genomic analysis supporting LUCID implementation for *B. cinerea* RNAi target discovery. This multi‐panel figure illustrates the integration of transcriptomic profiling and comparative genomics within the LUCID pipeline to identify candidate RNAi targets in *B. cinerea* . (a) Principal Component Analysis (PCA) reveals distinct expression profiles, enabling selection of genes upregulated during plant infection. (b) Volcano plot of differential expression analysis identifies 1151 significantly upregulated genes (log_2_FC > 1; adjusted *p*‐value < 0.05). (c) Heatmap highlights infection‐specific transcriptional responses, distinguishing infected samples from in vitro conditions. (d) Venn diagram‐style visualisation shows 3223 conserved orthogroups shared among six phytopathogenic fungi ( *B. graminis* , *B. cinerea* , *F. mangiferae*, *F. oxysporum*, *G. cichoracearum*, and *S. sclerotiorum*). (e) Gene Ontology (GO) enrichment analysis of infection‐induced genes reveals biological processes linked to pathogenicity. (f) GO enrichment of conserved orthogroups underscores functional categories relevant across fungal pathogens. Together, these analyses demonstrate LUCID's capacity to pinpoint conserved, infection‐specific genes as promising RNAi targets for fungal control strategies.

Applying the LUCID pipeline to *B. cinerea* enabled detailed analysis of gene expression and conservation during infection. Phase 1 filtering was crucial for identifying genes that are both infection‐specific and evolutionarily conserved across fungal pathogens. Principal Component Analysis (Figure 2a) revealed clear separation between in vitro controls and in vivo tomato infection samples, confirming robust transcriptomic shifts during host colonisation. A volcano plot (Figure 2b) identified 1151 significantly upregulated and 1470 downregulated genes, while the heatmap (Figure 2c) showed distinct expression profiles clustered by condition. In parallel, comparative proteome analysis across six phytopathogenic fungi identified 3223 conserved orthogroups (Figure 2d).

Functional enrichment analysis using Gene Ontology (GO) biological process terms was performed in upregulated genes. As shown in Figure 2e, genes were significantly enriched in several functional categories. Plant cell wall degradation was prominently represented, encompassing catabolic processes targeting cellulose, pectin, xylan, arabinan, and glucan, along with pathways involved in cell wall organisation. Other enriched processes included carbohydrate metabolism and transport, notably L‐arabinose metabolism, alongside fatty acid beta‐oxidation, proteolysis, transmembrane nutrient transport, and specialised metabolism such as L‐tryptophan catabolism to kynurenine. These findings suggest that *B. cinerea* extensively reprograms its transcriptome during infection to boost plant cell wall degradation, nutrient uptake, and energy production, key functions for effective host colonisation and pathogenesis.

Functional enrichment of the conserved orthogroups (Figure 2f) revealed common biological processes such as intracellular transport, protein folding and degradation, RNA and DNA processing, reproduction, and energy metabolism. These conserved processes reflect core biological functions maintained across diverse phytopathogenic fungi throughout their lifecycle, including infection. By intersecting expression and conservation datasets, we identified 225 candidate proteins that are consistently upregulated during infection and conserved across all six species, representing a foundational set of targets for RNAi‐based biofungicide development.

### 2.3 LUCID Pipeline Identified 13 CEPs in *B. cinerea*

**FIGURE 3:**
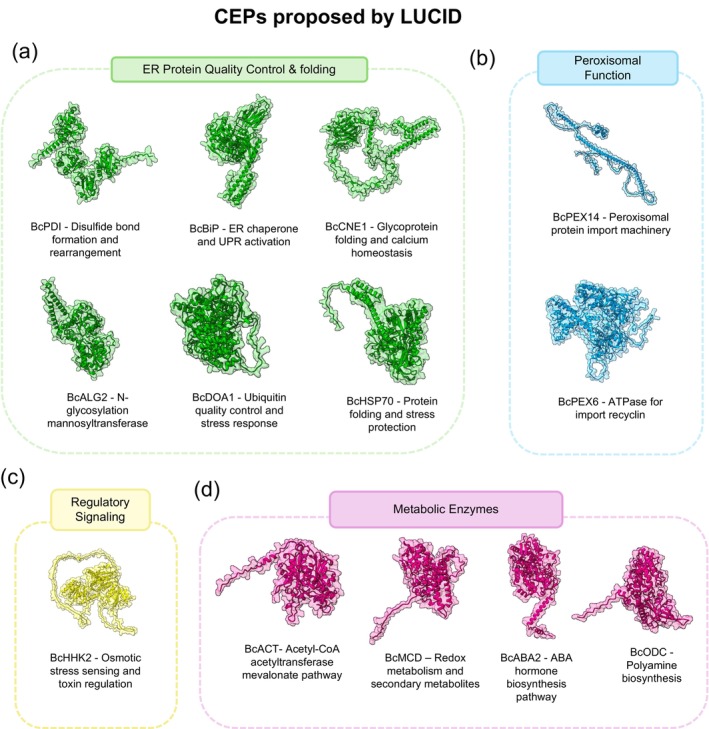
Functional categorization of Conserved Essential Proteins (CEPs) identified by LUCID in *B. cinerea* . Representative 3D protein structures and functional organisation of 13 CEPs critical for pathogenesis, grouped by cellular function and subcellular localization. (a) ER protein quality control and ERAD machinery comprising six proteins essential for protein folding, glycosylation, and degradation: BcPDI (protein disulfide‐isomerase), BcBiP (ER chaperone BiP), BcCNE1 (calnexin), BcHSP70 (heat shock protein 70), BcALG2 (mannosyltransferase), and BcDOA1 (ubiquitin system protein). (b) Peroxisomal proteins critical for organelle function: BcPEX14 (peroxisomal import machinery) and BcPEX6 (ATPase for import recycling). (c) Regulatory signalling proteins including BcHHK2 (histidine kinase) essential for osmotic stress sensing and toxin regulation. (d) Metabolic enzymes controlling key biosynthetic pathways: BcACT (acetyl‐CoA C‐acetyltransferase), BcMCD (short‐chain dehydrogenase), BcODC (ornithine decarboxylase), and BcABA2 (cytochrome P450 ABA2). Protein structures were obtained from the AlphaFold database or modelling.

The LUCID pipeline identified 13 Conserved Essential Proteins (CEPs) in *B. cinerea* through a homology‐based search against known pathogenesis‐related proteins. These CEPs span a range of cellular functions vital to *B. cinerea* pathogenicity and were classified into four major functional categories based on their subcellular localization and biological roles (Figure 3).

The largest functional group comprises ER‐localised protein quality control and folding machinery (Figure 3a), including six putative essential proteins implicated in the coordination of protein maturation. This group encompasses key molecular chaperones such as the heat shock protein HSP70 (BcHSP70), the ER‐resident chaperone BiP (BcBiP), and the glycoprotein‐specific chaperone calnexin (BcCNE1), all critical for maintaining protein homeostasis under infection stress. Additionally, this category includes the protein disulfide‐isomerase (BcPDI) essential for correct disulfide bond formation in secreted virulence factors, the mannosyltransferase ALG2 (BcALG2) required for N‐linked glycosylation of infection‐related proteins, and the ubiquitin system protein DOA1 (BcDOA1) that controls protein quality through targeted degradation pathways.

A second category encompasses peroxisomal proteins (Figure 3b) essential for organelle biogenesis and function, with PEX14 (BcPEX14) and PEX6 (BcPEX16) being critical for peroxisome protein import processes. These proteins are essential for β‐oxidation pathways, appressorium development, and cellular redox balance maintenance during host colonisation.

The third group consists of regulatory signalling proteins (Figure 3c) essential for pathogen adaptation to host environments. The histidine kinase HHK2 (BcHHK2) serves as a critical sensor for osmotic and oxidative stress conditions, coordinating cellular responses and regulating toxin production under specific metabolic conditions encountered during plant colonisation.

Finally, metabolic enzymes (Figure 3d) complete the identified targets, controlling key biosynthetic and catabolic pathways during infection. This includes the acetyl‐CoA C‐acetyltransferase (BcACT) involved in the mevalonate pathway and terpenoid biosynthesis, the short‐chain dehydrogenase (BcMCD) essential for redox metabolism and secondary metabolite production, the ornithine decarboxylase (BcODC) required for polyamine biosynthesis, and the cytochrome P450 monooxygenase ABA2 (BcABA2) crucial for abscisic acid hormone production that manipulates host defenses. These proteins are critical for β‐oxidation pathways, appressorium development, and cellular redox balance maintenance during host colonisation.

This functional diversity highlights how successful plant infection requires coordinated regulation across multiple essential cellular pathways, from protein quality control and stress adaptation to specialised metabolism and organelle function. Detailed information regarding the accession number, specific molecular functions, pathogenesis implications, and supporting literature for each CEP is provided in Table S1.

### 2.4 LUCID Pipeline Identified 17 CNAPs in *B. cinerea*

The LUCID pipeline identified 17 highly expressed Conserved Non‐Annotated Proteins (CNAPs) in *B. cinerea*. To infer their potential roles in pathogenesis, a two‐step annotation strategy was employed, integrating sequence homology searches with structural analysis. While sequence‐based homology successfully predicted putative functions for most CNAPs, BcUNC1, BcUNC2, and BcUNC3 were subjected to structural analysis using ProteinCartography (Avasthi et al. 2023). This approach revealed structural similarities to a mediator complex subunit, a cation‐transporting P‐type ATPase C‐terminal domain‐containing protein, and a transcription activator with a GCR1‐like domain, respectively, offering new insights into their potential roles in fungal pathogenesis.

**FIGURE 4:**
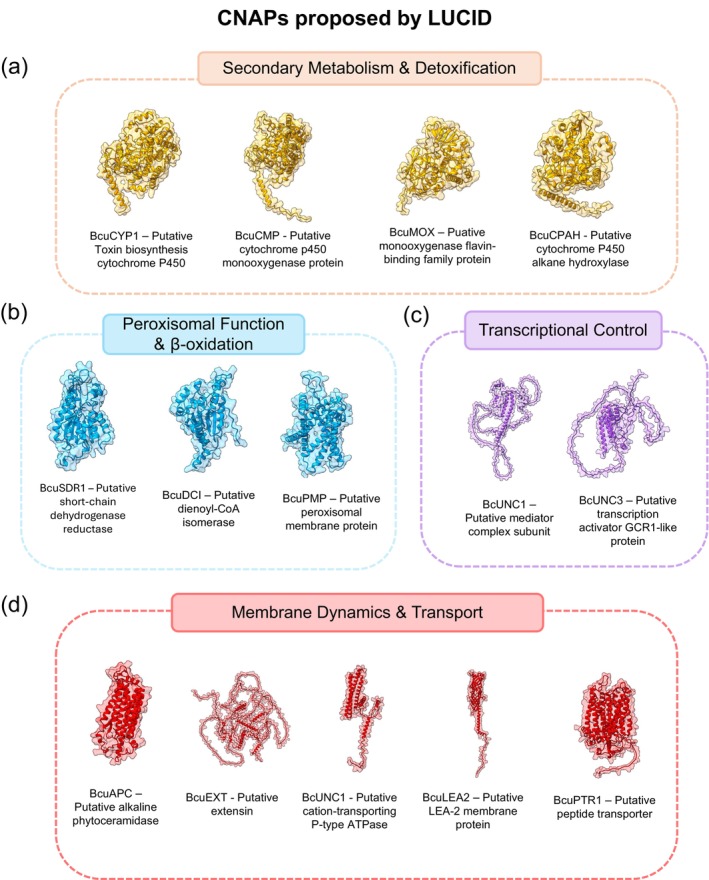
Functional categorization of Conserved Non‐Annotated Proteins (CNAPs) identified by LUCID in *B. cinerea* . Representative 3D protein structures and functional organisation of 17 CNAPs potentially critical for pathogenesis, grouped by predicted cellular function and subcellular localization. (a) Secondary metabolism and detoxification proteins comprising four proteins essential for metabolite production and host compound processing: BcuCYP1 (toxin biosynthesis cytochrome P450), BcuCMP (sterol metabolism monooxygenase), BcuCPAH (alkane hydroxylase for cuticle metabolism), and BcuMOX (FAD/NAD(P) redox monooxygenase). (b) Peroxisomal function and β‐oxidation proteins including six proteins critical for organelle integrity and fatty acid metabolism: BcuSDR1‐4 (short‐chain dehydrogenase/reductase enzymes), BcuDCI (dienoyl‐CoA isomerase), and BcuPMP (peroxisomal membrane protein). (c) Transcriptional control proteins including two regulatory proteins essential for gene expression coordination: BcUNC1 (mediator complex subunit) and BcUNC3 (transcription activator GCR1‐like). (d) Membrane dynamics and transport proteins featuring five proteins involved in cellular structure and transport processes: BcuAPC (alkaline phytoceramidase), BcuEXT (extensin cell wall organisation protein), BcUNC2 (cation‐transporting P‐type ATPase), BcuLEA2 (LEA‐2 membrane stabilisation protein), and BcuPTR1 (peptide transporter). Protein structures were obtained from the AlphaFold database or modelling.

CNAPs were organised into four main functional categories (Figure 4). The largest group comprises secondary metabolism and detoxification proteins (Figure 4a), including cytochrome P450 enzymes (BcuCYP1, BcuCMP, and BcuCPAH) and a FAD/NAD(P)‐binding protein (BcuMOX), all essential for metabolite production, host compound detoxification, and redox homeostasis during infection. A second significant group encompasses peroxisomal proteins (Figure 4b) critical for β‐oxidation and organelle function, comprising four short‐chain dehydrogenase/reductase enzymes (BcuSDR1‐4), a dienoyl‐CoA isomerase (BcuDCI), and a peroxisomal membrane protein (BcuPMP). The third group includes membrane dynamics and transport proteins (Figure 4d), featuring an alkaline phytoceramidase (BcuAPC), an extensin domain‐containing protein (BcuEXT), the cation‐transporting ATPase (BcUNC2), a LEA‐2 domain protein (BcuLEA2), and a peptide transporter (BcuPTR1), all involved in cellular structure maintenance and transport processes. Finally, transcriptional control proteins (Figure 4c) complete the identified targets, including the mediator complex subunit (BcUNC1) and the transcription activator (BcUNC3), essential for coordinating gene expression and cellular responses during host colonisation.

This functional diversity highlights how successful plant infection requires coordinated regulation across multiple cellular pathways, from specialised metabolism and organelle function to transcriptional control and membrane dynamics. Detailed information on accession numbers, predicted subcellular localizations, functional annotations, and pathogenesis implications for each CNAP is provided in Table S2.

### 2.5 LUCID Pipeline Enables the Generation of Highly Effective dsRNAs for Gene Silencing

To validate the LUCID workflow's utility in identifying targets for RNA interference (RNAi)‐based fungicides, we selected a subset of candidate genes for dsRNA‐mediated silencing experiments. From the CEPs group, seven proteins were randomly chosen to demonstrate the pipeline's capacity to rapidly identify RNAi targets. Additionally, five CNAPs were selected to explore novel mechanisms of action and assess the roles of uncharacterized proteins in pathogenesis. This CNAP subset included the three most challenging‐to‐annotate proteins (BcUNC1, BcUNC2, and BcUNC3) along with two randomly selected candidates. For each target, Phase 2 of the LUCID pipeline (dsRNA Design) was employed to generate optimal dsRNA molecules targeting regions with the highest density of effective siRNAs. Our modified SiFi21 analysis facilitated the identification of these regions and automated primer design for amplification, ensuring dsRNA constructs were of optimal size for fungal uptake. Full details of the Phase 2 outputs are provided in Supplementary File S1.

**FIGURE 5:**
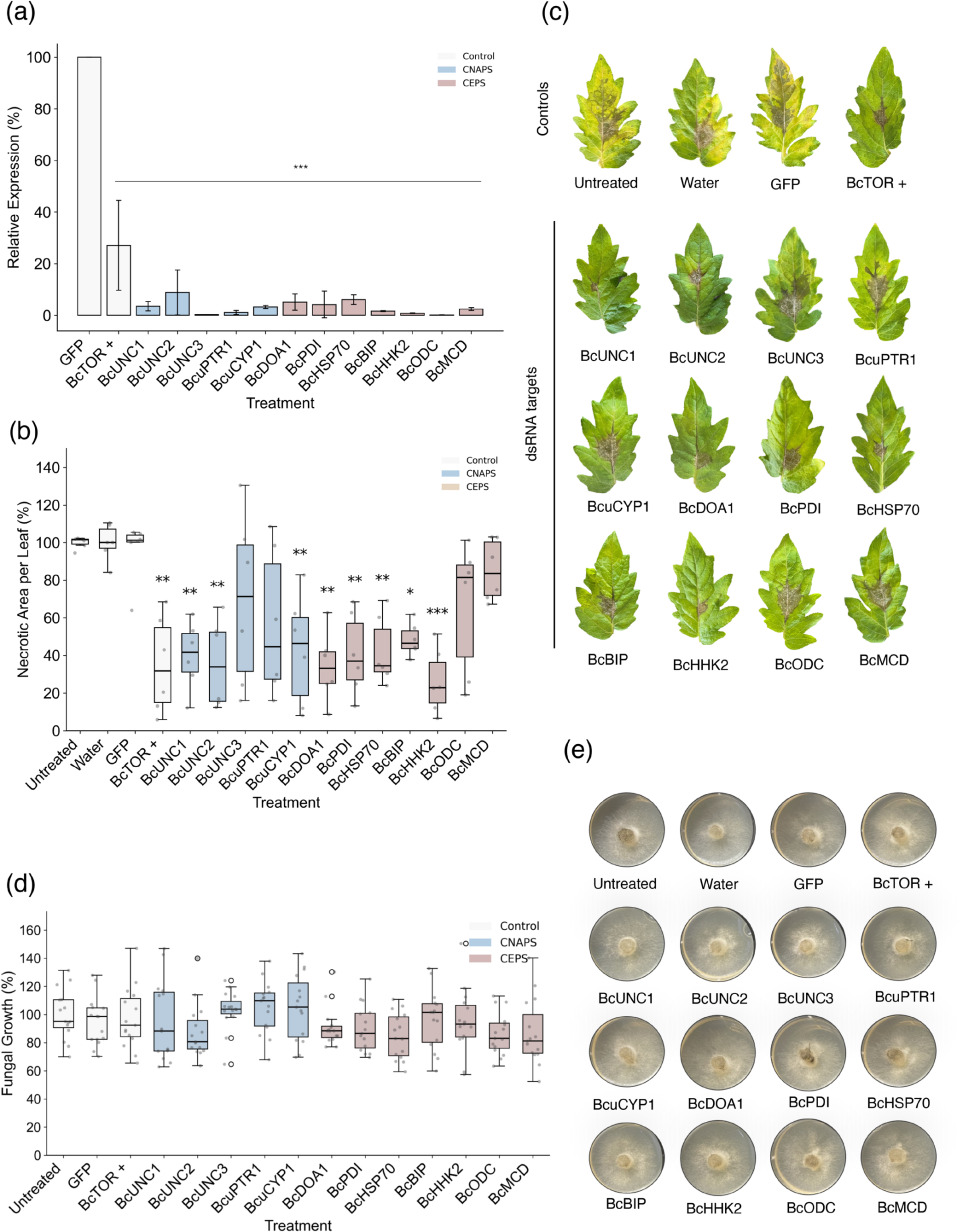
Experimental validation of LUCID‐predicted RNAi targets in the tomato–*Botrytis* pathosystem. This figure illustrates the efficacy of dsRNA‐mediated gene silencing targeting *B. cinerea* genes identified by the LUCID pipeline. (a) Relative expression levels of target genes in *B. cinerea* 24 h after dsRNA treatment, measured by RT‐qPCR. Significant downregulation confirms effective gene silencing compared to controls. (b) Quantification of necrotic leaf area (%) 72 h post‐infection across different dsRNA treatments. (c) Representative tomato leaf images showing disease symptoms following *B. cinerea* inoculation under various dsRNA treatments after 72 h. Controls include untreated leaves, GFP dsRNA (negative control), and TOR dsRNA (positive control). (d) Optical density‐based quantification of fungal growth in liquid PDB medium 48 h after dsRNA treatment. (e) In vitro growth assay of *B. cinerea* on PDA plates 48 h after dsRNA application. Statistical significance was assessed using one‐way ANOVA followed by Tukey's HSD test: *p* < 0.05 (*), *p* < 0.01 (**), and *p* < 0.001 (***). Individual data points were overlaid on box plots to show the distribution of the data. The boxes represent the interquartile range (IQR) with the median indicated by a horizontal line, and whiskers extend to 1.5 times the IQR. Gene expression data is presented as bar graphs with error bars indicating mean ± SEM.

Quantitative RT‐PCR analysis revealed significant gene silencing across all targets compared to the GFP dsRNA negative control (Figure 5a). BcTOR, a master regulator of cell growth and proliferation used as a positive control for RNAi, showed a reduced expression level of 27.08%. For CEPs, expression levels were similarly suppressed: BcDOA1 (5.10%), BcPDI (4.17%), BcHSP70 (6.11%), BcBIP (1.63%), BcHHK2 (0.81%), BcODC (0.12%), and BcMCD (2.45%). Among CNAPs, BcUNC1, BcUNC2, and BcUNC3 exhibited expression levels of 3.55%, 8.85%, and 0.26%, respectively. BcuPTR1 and BcuCYP1 were reduced to 1.12% and 3.27%. These results confirm robust silencing, with most targets reduced to below 10% of control levels.

*In planta* assays revealed varying degrees of disease suppression following dsRNA treatment, measured as percentage of disease development relative to negative control (Figure 5b,c). BcTOR, serving as a positive control, reduced disease to 34.94%. Among CEPs, BcHHK2 showed the strongest effect, reducing disease to 26.09%, outperforming the control. BcDOA1 was similarly effective (34.14%), while BcPDI and BcHSP70 also significantly reduced virulence (40.51% and 42.17%, respectively). BcMCD showed a more moderate impact (85.17%). For CNAPs, BcUNC2 was most effective (35.66%), followed by BcUNC1 (40.02%) and BcuCYP1 (42.96%). BcuPTR1 and BcUNC3 showed moderate reductions (56.44% and 69.19%). These effects were clearly visible in tomato leaf infection assays (Figure 5c). To assess whether dsRNA application affected general fungal growth, we conducted in vitro experiments in both liquid (PDB) media and solid (PDA). No significant changes in growth were observed across treatments (Figure 5d,e), indicating that the observed reductions in disease development were specifically due to interference with pathogenicity‐related genes rather than general growth inhibition.

### 2.6 Structural Characterisation and Functional Analysis of a Putative Mediator Complex Subunit as a Pathogenicity Target

**FIGURE 6:**
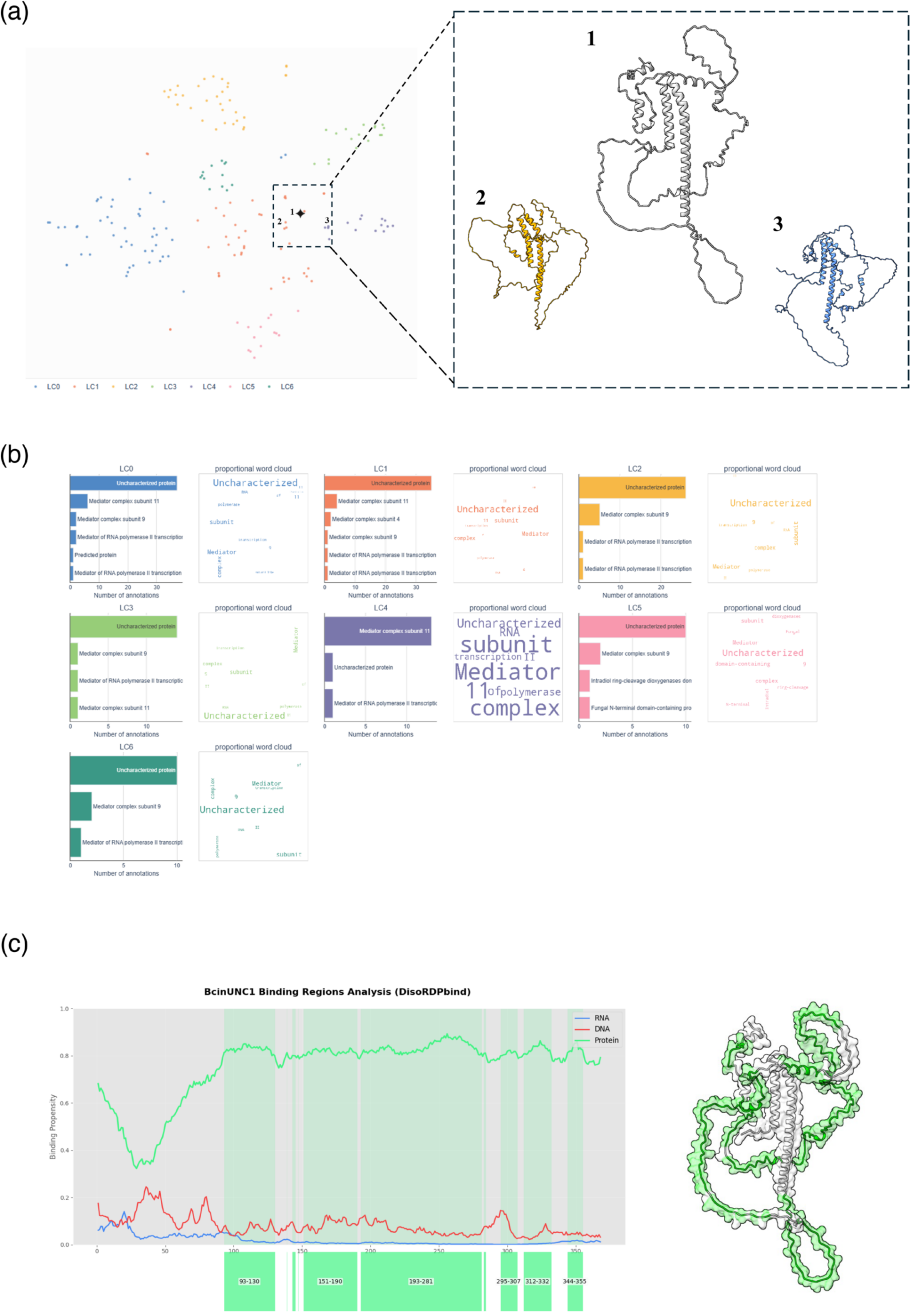
Structural and functional analysis of BcUNC1. This figure illustrates the structural context and predicted functional properties of the conserved non‐annotated protein BcUNC1. (a) Protein space visualisation generated by ProteinCartography, displaying Leiden clusters in distinct colours. The inset highlights the local protein neighbourhood surrounding BcUNC1 (white, 1), alongside its two closest annotated structural analogs: Mediator complex subunit 9 (orange, 2) and mediator complex subunit 11 (blue‐purple, 3). (b) Semantic analysis of functional annotation distributions across Leiden clusters LC0–LC6. Each panel includes bar charts and word clouds representing the frequency of associated biological functions. (c) Binding propensity profile of BcUNC1 disordered regions. The line plot shows predicted binding probabilities for RNA (blue), DNA (red), and proteins (green) along the amino acid sequence. On the right, the 3D structure of BcUNC1 is shown with predicted protein‐binding regions highlighted in green. Protein structure was obtained from AlphaFold database or modelling.

Although BcUNC2 exhibited the greatest reduction in *B. cinerea* pathogenicity, BcUNC1 was selected for detailed structural and functional analysis due to its superior silencing efficiency and consistent experimental outcomes (Figure 5b). Silencing of BcUNC1 via dsRNA resulted in a 60% decrease in disease development, indicating a significant role in pathogenesis. To investigate the molecular basis of this phenotype, structural analysis was conducted using ProteinCartography and AlphaFold predictions (Figure 6a,b). The protein structure revealed a combination of ordered α‐helical regions characteristic of mediator complex subunits, alongside extensive Intrinsically Disorder Regions (IDR). Predictions using DisoRDPbind (Figure 6c) indicated that these disordered regions are likely involved in protein–protein interactions, consistent with their role in assembling large macromolecular complexes such as the mediator complex.

**FIGURE 7:**
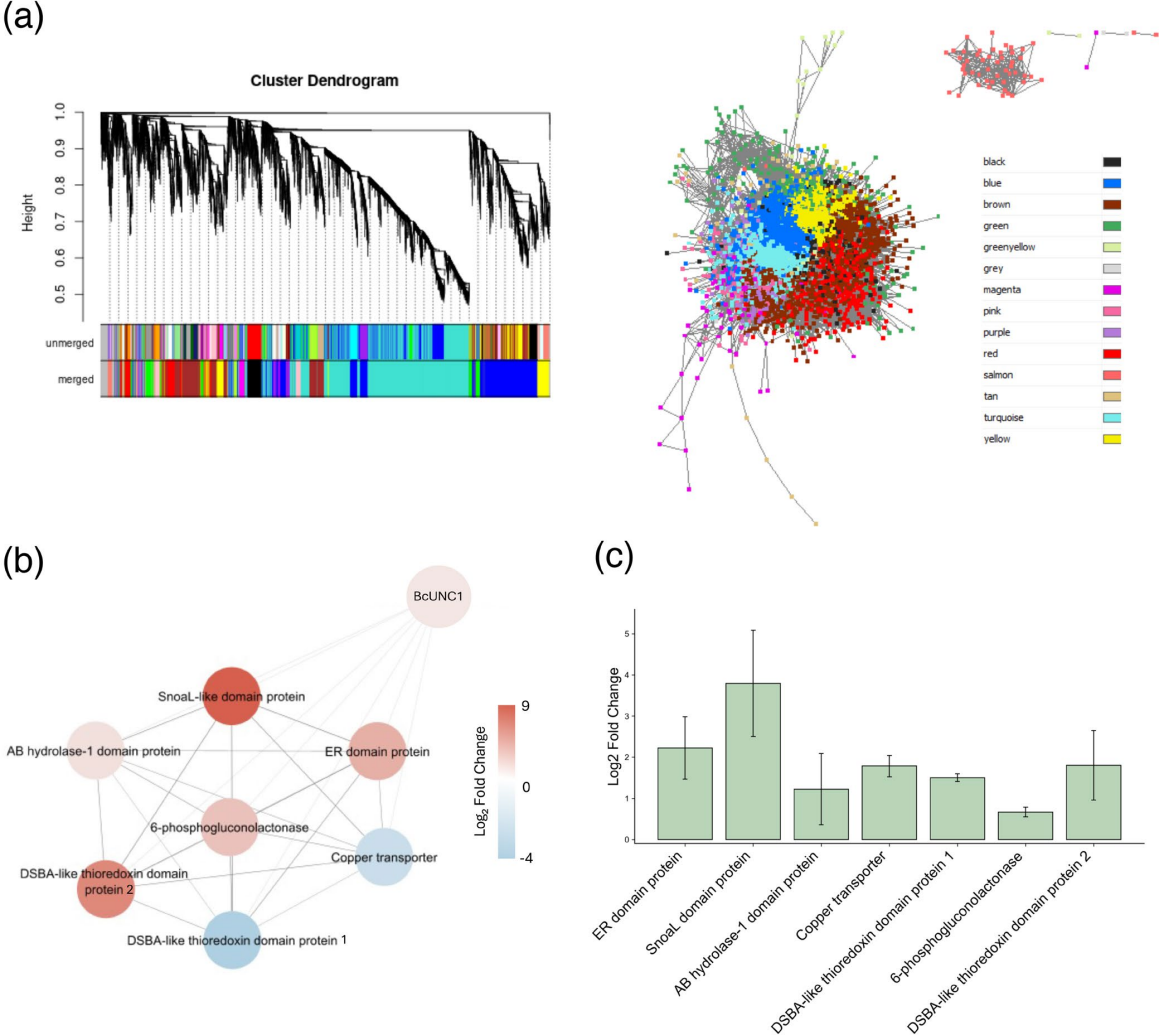
Co‐expression network analysis of *B. cinerea* transcriptome highlighting CNAP BcUNC1. This figure presents a Weighted Gene Co‐expression Network Analysis (WGCNA) of *B. cinerea* transcriptomic data, with emphasis on the conserved non‐annotated protein BcUNC1. (a) Hierarchical clustering dendrogram (top left) showing gene modules identified by WGCNA, with colour‐coded assignments before and after merging similar modules. Network visualisation (top right) displays gene connections with topological overlap (TOM) > 0.1, with node colours corresponding to module membership. (b) Subnetwork centred on mediator complex subunit (BcUNC1) within the brown module, illustrating its closest co‐expressed neighbours. Node colours reflect log_2_ fold‐change during infection: Red for upregulated genes, blue for downregulated. (c) RT‐qPCR analysis of genes co‐expressed with BcUNC1 in infected tomato plants treated with BcUNC1 dsRNA versus control dsRNA. Expression changes are shown as log_2_ fold‐change, with data presented as mean ± standard error from three independent biological replicates.

To further explore BcUNC1's functional role, a co‐expression network analysis was performed integrating differential expression data from infection versus non‐infection conditions (Figure 7a). The resulting network revealed a group of co‐expressed genes contributing to a coordinated virulence programme (Figure 7b). Notably, several secondary metabolism enzymes were strongly co‐expressed with BcUNC1, including: an enoyl reductase (ER) domain‐containing protein, upregulated during infection and localised to the cytoplasm, which catalyses double bond reduction in polyketide and fatty acid biosynthesis, contributing to phytotoxin production. A SnoaL‐like domain‐containing cyclase, also upregulated, involved in the synthesis of complex cyclic secondary metabolites. An extracellular AB hydrolase‐1 domain‐containing lipase/esterase, which degrades structural plant lipids to facilitate penetration of cuticles and cell membranes. The network also revealed differential regulation of redox homeostasis components: a cytoplasmic DSBA‐like thioredoxin domain‐containing protein 1 was downregulated during infection, suggesting reduced thiol‐disulfide exchange activity in the cytoplasm. In contrast, a mitochondrial DSBA‐like thioredoxin domain‐containing protein 2 was upregulated, indicating a compartmentalised redox strategy. A cytoplasmic 6‐phosphogluconolactonase, upregulated during infection, catalyses a key step in the oxidative phase of the pentose phosphate pathway, generating NADPH for biosynthesis and redox balance. An extracellular copper transporter, downregulated during infection, regulates copper availability for antioxidant enzymes and redox signalling. Detailed descriptions of these co‐expressed proteins and their potential roles in pathogenesis are provided in Table S3.

To validate the regulatory role of BcUNC1 on the genes identified in the co‐expression network, qPCR analysis was performed comparing gene expression in infected plants treated with control dsRNA versus dsRNA targeting BcUNC1. BcUNC1 silencing resulted in significant upregulation of most analysed genes compared to the control (Figure 7c). The genes showing the highest expression increases were those encoding the SnoaL domain protein (3.79 log2 fold‐change) and the ER domain protein (2.22 log2 fold‐change). The DSBA‐like thioredoxin domain protein 2 showed 1.80 log2 fold‐change, while the copper transporter exhibited 1.79 log2 fold‐change. The DSBA‐like thioredoxin domain protein 1 displayed 1.50 log2 fold‐change, and the AB hydrolase‐1 domain protein showed 1.22 log2 fold‐change. The 6‐phosphogluconolactonase showed the smallest change (0.67 log2 fold‐change).

These results suggest that BcUNC1 functions as a fine‐tuning transcriptional regulator that coordinates controlled expression of virulence‐associated genes during infection, rather than simply acting as a direct activator.

## 3 Discussion

The development of dsRNA‐based control strategies for phytopathogenic fungi faces significant challenges in target identification and validation, often requiring years of empirical testing to identify effective silencing targets (Keerthana and Koteeswaran 2024). Traditional approaches rely on candidate gene selection based on limited functional information, leading to lengthy development cycles and frequent failures in achieving sufficient pathogen control. To address these bottlenecks, we introduce LUCID, a novel computational pipeline that identifies optimal targets for RNAi‐based control of phytopathogenic fungi.

In our implementation of LUCID on *B. cinerea* , we used RNA‐seq data from early tomato leaf infection (24 hpi) to capture genes active during initial host colonisation. This experimental design is critical, as the timing, host species, and infection conditions of transcriptomic sampling directly influence which genes emerge as infection‐associated candidates, and users should tailor their RNA‐seq experiments to the specific pathosystem and disease stage most relevant to their control objectives. Functional enrichment analysis of the resulting upregulated genes in *B. cinerea* revealed significant overrepresentation of biological processes directly linked to fungal pathogenicity. Plant cell wall degradation processes were prominently represented, including cellulose, pectin, xylan, and arabinan catabolic processes, which are essential for host penetration and nutrient acquisition. The enrichment in carbohydrate metabolism and transport, fatty acid metabolism, and specialised metabolism pathways further underscores the metabolic reprogramming that occurs during infection. These findings align with established knowledge of fungal pathogenicity mechanisms, where the ability to degrade host cell walls and efficiently utilise host‐derived nutrients is crucial for successful colonisation (Kubicek et al. 2014). In parallel, comparative proteomics identified conserved proteins shared across multiple phytopathogenic fungi, enriched in functions such as protein homeostasis, vesicle‐mediated transport, and energy metabolism. The conservation of these processes across diverse fungal pathogens suggests their essential role in the pathogenic lifestyle (Giraldo et al. 2013; Liu et al. 2025). Orthology‐based conservation analyses can be affected by proteome annotation quality and completeness, and both broad and partial conservation may reflect housekeeping rather than virulence‐specific roles. For this reason, LUCID integrates these data through multi‐layered filtering that requires pathogen‐wide conservation, infection‐induced upregulation, and homology to experimentally validated virulence factors. This approach enriches for functionally relevant pathogenicity related proteins, as supported by saprophytic‐growth assays. Importantly, infection‐associated expression is inherently time‐dependent: early sampling captures genes suited for preventive or early‐intervention strategies, whereas later infection stages highlight targets more appropriate for curative applications. Although constitutively expressed essential genes could, in principle, support preventive treatments, they carry higher risks of affecting conserved pathways. Incorporating multiple infection stages or targeted time‐course analyses for promising candidates may therefore refine target selection and optimise application windows.

The CEPs branch demonstrated remarkable efficacy with 71.4% of tested candidates showing disease reduction comparable to or better than the positive control. The effectiveness of our approach is particularly exemplified by BcHhk2, which reduced disease development to only 26.09%. This histidine kinase group V protein plays a crucial role in signal transduction pathways, contributing to virulence through osmotic and oxidative stress adaptation and toxin production (Escobar‐Niño et al. 2021). Similarly, the significant virulence reduction observed with BcDOA1 and BcPDI highlights the critical importance of protein quality control processes during infection, consistent with findings from other pathosystems (Stolf et al. 2011; Noman et al. 2023; Wang et al. 2024). A particularly noteworthy observation from our study is the prominence of peroxisome‐associated proteins among both CEPs and CNAPs groups. Within the CEPs, the identification of BcPEX14 and BcPEX6 underscores the critical role of peroxisomes in fungal pathogenicity. Peroxisomes are increasingly recognised as critical organelles in fungal‐plant interactions, contributing to fatty acid metabolism, detoxification of reactive oxygen species, and secondary metabolism (Imazaki et al. 2010; Wei et al. 2013; Falter and Reumann 2022). This aligns with previous studies in *Magnaporthe oryzae*, where peroxisomal function was shown to be essential for appressorium‐mediated host penetration and adaptation to oxidative stress during infection (Li et al. 2017; Wang et al. 2019; Chen et al. 2024).

One of the most impactful outcomes of our study is the identification of novel virulence factors through the CNAPs branch. This approach builds upon recent advances in a work that successfully identified novel target candidates in *Podosphaera xanthii* for controlling powdery mildews through spray‐induced gene silencing (Ruiz‐Jiménez et al. 2021). By combining structural predictions using ProteinCartography (Avasthi et al. 2023), which employs state‐of‐the‐art protein language models (PLMs) to identify functional relationships beyond traditional sequence similarity approaches (Lin et al. 2023), we have inferred functional annotations for previously uncharacterized proteins with implications in pathogenesis. Notably, BcUNC1, BcUNC2 and BcuCYP1 showed strong silencing effects, reducing disease development to 40.02%, 35.66% and 42.96% respectively. These results validate our approach of targeting conserved yet previously uncharacterized proteins as an effective strategy for identifying novel pathogenicity factors. Importantly, in vitro PDA assays showed that silencing did not affect normal pathogen growth, strongly supporting that these are pathogenesis‐specific genes identified through our targeted strategies, which should significantly reduce off‐target effects on beneficial non‐pathogenic fungi.

The selection of BcUNC1 was motivated by the critical role of transcriptional reprogramming in coordinating the expression of pathogenesis arsenals during infection (Moran et al. 2019; John et al. 2021). The structural features of this protein combine ordered α‐helical regions similar to Mediator complex subunits 9 and 11 (Verger et al. 2019), and extensive intrinsically disordered regions that facilitate flexible protein–protein interactions (Peng and Kurgan 2015). Proteins with disordered regions are of particular interest in pathogenesis research, as these regions have been shown to be significantly associated with virulence in fungi (Chepsergon and Moleleki 2023). The evolutionary expansion of intrinsically disordered regions within Mediator complex subunits has been particularly pronounced in more complex organisms, with these flexible domains conferring enhanced regulatory capabilities and enabling dynamic protein–protein interactions essential for transcriptional control (Nagulapalli et al. 2016). Studies of Mediator complex components in fungal pathogens support this hypothesis, as they have demonstrated their crucial role in integrating regulatory signals and facilitating recruitment of transcriptional machinery to virulence‐related gene promoters (Bourbon 2008).

Co‐expression network analysis further revealed that BcUNC1 is tightly linked to diverse virulence‐related processes. These include secondary metabolism enzymes such as an enoyl reductase involved in host‐specific phytotoxin biosynthesis (Yang et al. 1996; Lysøe et al. 2006; Li et al. 2020) and a SnoaL‐like domain protein involved in phytotoxic compound synthesis (Ferrara et al. 2021). Co‐expression was also observed with an AB hydrolase‐1 domain protein that degrades plant lipids, aiding in host barrier penetration and nutrient acquisition (de Torres Oliveira et al. 2024), and metabolic enzymes including a 6‐phosphogluconolactonase that supports high energy demands and redox homeostasis during infection (Wilson et al. 2007). Additionally, the network included redox‐related proteins such as DSBA‐like thioredoxins and a copper transporter affecting redox balance in host‐pathogen interactions (Shahid et al. 2014; Hammerstad and Hersleth 2021; Ray and Rappleye 2022; Tian et al. 2023). Functional validation revealed a regulatory role for BcUNC1: silencing this gene led to significant upregulation of most co‐expressed targets, suggesting that BcUNC1 functions not as a direct activator but as a transcriptional balancer. This regulatory behaviour aligns with known dual roles of Mediator subunits in fungi, which can act as activators or repressors depending on cellular context (Cao et al. 2016; Zhou et al. 2022). Taken together, these findings support the model that BcUNC1 orchestrates the balanced expression of metabolic, redox, and secretion‐related programmes essential for successful infection.

The dual‐branch strategy employed in LUCID workflow proved highly effective in identifying silencing targets through a comprehensive approach that systematically integrates transcriptomic and comparative genomic data. This success rate significantly exceeds the performance of traditional approaches (McLoughlin et al. 2018; Chen, Shi, et al. 2025). Also, the integration of dsRNA design generates primers that amplify regions producing highly effective dsRNAs for silencing, as demonstrated by the consistent transcript silencing achieved across all tested targets. Furthermore, to address off‐target concerns, LUCID incorporates off‐target prediction against user‐provided non‐target transcriptomes and chimeric dsRNA design through dsRNAmax (Fletcher et al. 2025). This enables computational assessment of cross‐reactivity with host plants, beneficial fungi, or pollinators, mitigating risks that conserved sequences in phytopathogenic fungi may affect non‐target organisms. The integration of dsRNAmax also facilitates the design of multi‐target chimeric dsRNAs from validated candidates, enabling broad‐spectrum formulations while maintaining sequence specificity.

Concentration optimization is critical for developing competitive products. Our *in planta* assays used 50 ng/μL, consistent with recent SIGS validations in *B. cinerea* (Capriotti et al. 2024; Qiao et al. 2023). However, it has also been described that dsRNA above 10 ng/μL can trigger sequence‐independent PAMP responses (Höfle et al. 2025). For this reason, although our results support primarily RNAi‐mediated effects, field applications would benefit from concentration optimization to balance efficacy and cost‐effectiveness.

With RNAi‐based products already approved and others in development, biotechnology companies need robust computational tools to remain competitive in the evolving plant health landscape (De Schutter et al. 2022). In a sector where only a small fraction of candidate compounds or technologies progress from early‐stage discovery to commercial deployment, LUCID offers a powerful solution to reduce R&D risk by focusing experimental efforts on the most promising computationally identified targets (Gathman et al. 2025).

## 4 Experimental Procedures

### 4.1 LUCID—Workflow

LUCID (Locating Uncovered, Conserved and Indispensable for Pathogenicity Determinants) is a novel computational pipeline designed to identify optimal targets for RNA interference‐based control strategies in phytopathogenic fungi. By combining transcriptomic profiles with comparative genomic analysis, LUCID identifies genes that are both highly active during infection and conserved among selected fungal species. The pipeline operates in two phases: Phase 1 (Target Selection) leverages transcriptomic and genomic data to uncover candidate genes for silencing. Phase 2 (dsRNA Design) focuses on crafting double‐stranded RNA molecules tailored for effective gene knockdown, ensuring high specificity and silencing potency within fungal systems. The complete source code, datasets, and usage guidelines are publicly accessible at https://github.com/Brunxi/LUCID.

#### 4.1.1 Phase 1: Target Selection

LUCID's workflow consists of several interconnected steps: (1) Data preparation, including organisation of genome assemblies and annotations, proteomes, and RNA‐seq datasets; (2) Orthology analysis to identify conserved proteins across multiple fungal species of interest; (3) Gene expression analysis, depending on the fungal lifestyle, either TPM‐based quantification (for obligate biotrophs) or differential expression analysis (for non‐obligate biotrophs) is applied to assess transcriptional activity during infection; and (4) Target identification through integration of expression data, conservation patterns, and homology searches against a curated database of known pathogenicity factors. This approach categorises potential targets into two groups: Conserved Essential Proteins (CEPs), which are conserved, highly expressed proteins with known functions essential for pathogenicity in other phytopathogens, and Conserved Non‐Annotated Proteins (CNAPs), which represent novel conserved proteins with high expression levels during infection but lacking functional annotation.

#### 4.1.2 Phase 2: dsRNA Design

The second stage of the LUCID pipeline focuses on designing optimal double‐stranded RNA (dsRNA) molecules to silence the gene targets identified during Phase 1. This phase utilises both the selected transcripts and the full transcriptome of the target fungal pathogen as input. To achieve precise and effective gene silencing, LUCID integrates a customised version of the siFi21 software (Lück et al. 2019), a widely adopted tool for predicting functional small interfering RNAs (siRNAs). For each transcript, the pipeline conducts a detailed analysis to pinpoint the most suitable silencing regions by: (1) Predicting siRNAs, evaluating all possible 21‐nt siRNA windows along the transcript based on sequence characteristics, thermodynamic stability, and target site accessibility; (2) Identifying high‐density windows: the algorithm employs a sliding window approach to scan the transcript for regions between 200 and 400 bp (Höfle et al. 2020), selecting the window with the highest density of effective siRNAs, defined as those meeting both efficiency criteria and accessibility thresholds (target site accessibility > 0.1). The final dsRNA length is determined by that specific region showing maximal siRNA density; (3) Visualising siRNA distribution, producing graphical outputs that map siRNA effectiveness across the transcript, clearly marking the optimal silencing zone; (4) Generating JSON output, compiling a structured file containing siRNA sequences, accessibility metrics, and all relevant parameters for downstream use; (5) Designing primers, automatically generating primers with Primer3 (Untergasser et al. 2012) to amplify the region selected in step 2. This streamlined and automated process equips researchers with all the necessary data to synthesise potent dsRNA molecules, precisely targeting the most accessible and effective regions of fungal transcripts.

#### 4.1.3 Phase 3: Off‐Target Prediction and Chimeric dsRNA Design

The third phase evaluates potential off‐target effects and enables multi‐target dsRNA construction. For off‐target assessment, the pipeline extracts all 21‐nt siRNAs from each designed dsRNA and performs BLAST searches against the non‐target transcriptomes to identify potential matches. Hits with high sequence similarity are flagged for user review. Additionally, this phase integrates dsRNAmax (Fletcher et al. 2025) to design chimeric dsRNAs. Users can input multiple validated target sequences, and dsRNAmax generates chimeric constructs that combine these targets while minimising sequence redundancy and maintaining RNAi efficacy.

### 4.2 Data Sources and Preparation: RNA‐Seq, Genomes, Proteomes, and Virulence Databases

To identify RNAi targets in *B. cinerea* , transcriptomic data were retrieved from NCBI for strain B05.10 under two conditions: tomato infection (SRR6924534–SRR6924536) and in vitro growth (SRR6924547–SRR6924549). These datasets enabled comparative analysis between parasitic and saprophytic lifestyles. The reference genome used for alignment and annotation was obtained from Ensembl Fungi (assembly ASM361119v1). For conservation analysis, proteomes from six phytopathogenic fungi were sourced from Ensembl Fungi: *Blumeria graminis* (EF2), *B. cinerea* (ASM83294v1), *Fusarium mangiferae* (version 1), *F. oxysporum* (FO2), *Golovinomyces cichoracearum* (ASM361119v1), and *Sclerotinia sclerotiorum* (ASM14694v1). To facilitate homology‐based identification of Conserved Essential Proteins (CEPs), a curated pathogenicity database was assembled by integrating multiple resources: PHI‐base (Urban et al. 2020), DEG (Luo et al. 2021), VEuPathDB (Alvarez‐Jarreta et al. 2024), and DFVF (Lu et al. 2012). Redundant sequences with greater than 40% identity were filtered using DIAMOND (v2.1.11) (Buchfink et al. 2015).

### 4.3 Computational Methods for Orthology, Transcriptomics, and Homology Analyses

For orthology analysis, OrthoFinder (v2.5.5) (Emms and Kelly 2019) was used to identify conserved protein families across the selected fungal species. We specifically focused on orthogroups containing proteins present in all six phytopathogenic. For transcriptomics analysis, we utilised in R (v4.1.2) the Rsubread package (v2.8.2) (Liao et al. 2013) for read alignment and quantification, followed by differential expression analysis with DESeq2 (v1.34.0) (Love et al. 2014). Genes were considered significantly upregulated during infection when they exhibited a log2 fold change > 1 and an adjusted *p*‐value < 0.05. Homology searches against our curated database of essential and virulence‐required genes were performed using DIAMOND (v2.1.11) (Buchfink et al. 2015). Strict filtering criteria, retaining only proteins with > 45% sequence identity and a bitscore > 50 to identify CEPs with high confidence were applied.

### 4.4 Annotation Tools

To identify Conserved Non‐Annotated Proteins (CNAPs), we focused on conserved, upregulated proteins that lacked functional annotation or characterisation in UniProt entries. Initial functional predictions were conducted using large‐scale BLAST searches, enabling tentative inference based on sequence similarity. Proteins that remained uncharacterized after these analyses were further examined using ProteinCartography (Avasthi et al. 2023), a tool that leverages structural information rather than sequence data. This structure‐based approach is particularly advantageous, as protein folding patterns are typically more conserved than amino acid sequences and often provide stronger functional clues, even when sequence homology is low. Predicted subcellular localization for all proteins was obtained using DeepLoc 2.0 (Thumuluri et al. 2022), and 3D structures were predicted using AlphaFold2 (Abramson et al. 2024). Functional enrichment analysis was performed using DAVID (Sherman et al. 2022). For protein–protein interactions involving intrinsically disordered regions, we used DisoRDPbind (Peng and Kurgan 2015).

### 4.5 Plants, Microbes, Nucleic Acid Extraction and cDNA Synthesis

Tomato ‘Moneymaker’ plants (*Solanum lycopersicum*) were cultivated at 24°C under 16 h light/8 h darkness for dsRNA assays. *B. cinerea* B05.10 was maintained on PDA. *E. coli* DH5α was cultured at 37°C in LB medium with ampicillin (100 μg m L^−1^) for RNAi vector work. Infected tomato leaves were frozen in liquid nitrogen, stored at −80°C, and pulverised. Total RNA was extracted using TRI Reagent (Sigma‐Aldrich), eluted in DEPC‐treated water, and stored at −80°C. RNA concentration was determined using NanoDrop 2000 (Thermo Fisher Scientific). cDNA synthesis was performed using Superscript III reverse transcriptase with random primers following the manufacturer's protocols.

### 4.6 In Vitro Production of dsRNA

For dsRNA production, target sequences were cloned into plasmids with opposing T7 RNA polymerase promoters, followed by one‐step PCR amplification with flanking T7 sequences and in vitro transcription. *B. cinerea* gene fragments and a 379 bp GFP control fragment were amplified from cDNA using Phusion High‐Fidelity DNA polymerase (Thermo Fisher Scientific) with Primer3‐designed primers (Untergasser et al. 2012). PCR conditions: 98°C/30 s, then 35 cycles of 98°C/10 s, 60°C/30 s, 72°C/30 s, final extension 72°C/7 min. PCR products were digested with FastDigest KpnII and BglII (Thermo Fisher Scientific) and ligated into pL4440 vector. Plasmids were propagated in *E. coli* DH5α and verified by PCR, digestion, and sequencing. Inserts flanked with T7 promoters were amplified using T7‐F/T7‐R primers, purified, and used for in vitro transcription with MEGAScript RNAi kit (Invitrogen). dsRNA concentration was determined using NanoDrop 2000, and integrity confirmed on 1.5% agarose gels.

### 4.7 dsRNA‐Mediated Gene Silencing Assays

In planta dsRNA‐mediated gene silencing was performed following previous detached leaf assays (McLoughlin et al. 2018) with minor modifications. Leaves from 4‐week‐old plants were placed adaxial side up in petri dishes containing 0.5% water agar. A 5 μL solution containing 500 ng of dsRNA was mixed with 5 μL of *B. cinerea* spores prepared in Gamborg's B‐5 minimal medium (GB5: 3.05 g L^−1^, 10 mM KH_2_PO_4_, 25 mM glucose, pH 5.5; 1 × 10^4^ spores mL^−1^) and applied to the leaf surface. *B. cinerea* TOR gene served as a positive control (Xiong et al. 2019; Escobar‐Niño et al. 2021), while GFP dsRNA was used as a negative control (Ray et al. 2022). The plates were sealed with parafilm and incubated at 20°C with a 16 h light/8 h dark photoperiod at 100% relative humidity. Disease symptoms were evaluated by measuring lesion diameter using ImageJ (Schneider et al. 2012) software at 72 h post‐inoculation (hpi). Water and GFP‐dsRNA were used as controls. Each experiment included at least six biological replicates per treatment. Statistical analyses were performed using Python (v3.12) with statsmodels (v0.14.1) and scipy (v1.12.0) packages. The significance of differences in necrotic area per leaf between treatments was assessed using one‐way analysis of variance (ANOVA). Post hoc comparisons between treatments and water control were conducted using Tukey's Honestly Significant Difference (HSD) test.

In vitro assays were performed following previous protocols with modifications (Wang et al. 2016). For solid media assays, PDA plates were prepared and 50 μL drops containing dsRNA (20 ng/μL) were applied and evenly distributed across the surface using glass beads. After absorption of the dsRNA solution, discs of growing *B. cinerea* mycelium (5 mm diameter and 2 mm thickness; 5 × 10^5^ spores/mL) were made, placed on PDA plates and incubated for 48 h at 24°C. For liquid assays, spores were incubated in 1/2 PDB supplemented with dsRNA at a final concentration of 20 ng/μL and incubated for 48 h at 20°C and agitation (130 rpm). Growth inhibition was evaluated and the significance of differences in mycelial growth between treatments was assessed using one‐way analysis of variance (ANOVA). Post hoc comparisons between treatments and water control were conducted using Tukey's Honestly Significant Difference (HSD) test.

### 4.8 Quantitative Reverse Transcription RT‐qPCR

Gene expression analysis in *B. cinerea* was performed using RT‐qPCR. Primers were designed with Primer3 software (Table S4), specifically excluding the regions targeted by dsRNA to prevent amplification bias. To evaluate the silencing effect of dsRNA treatments, cDNA was synthesised from RNA extracted from *B. cinerea* ‐infected tomato leaf discs collected 24 h post‐inoculation (hpi). The *B. cinerea* ubiquitin gene *Bcsmt3* (Bcin11g03430) was used as the internal reference for normalisation (Ren et al. 2017). RT‐qPCR assays were carried out on an Applied Biosystems StepOnePlus Real‐Time PCR System (Thermo Fisher Scientific) using KAPA SYBR FAST reagents (Kapa Biosystems), following the manufacturer's protocol. Thermal cycling conditions included an initial enzyme activation at 95°C for 3 min, followed by 40 cycles of 95°C for 3 s and 56°C for 20 s. Each reaction was run in triplicate, both technically and biologically. Post‐amplification, product sizes were verified via electrophoresis on 2% agarose gels, and melting curve analysis was conducted to confirm specificity. Relative gene expression was calculated using the 2^−ΔΔ*Ct*^ method (Livak and Schmittgen 2001), and relative expression as log2fc was calculated as −ΔΔ*Ct*. The experimental design included three biological and three technical replicates; standard deviation calculations were performed by independently calculating 2^−ΔΔ*Ct*^ or −ΔΔ*Ct* values for each biological replicate.

### 4.9 Co‐Expression Network Construction

To explore gene co‐expression patterns in *B. cinerea* , a Weighted Gene Co‐expression Network Analysis (WGCNA) was performed using RNA‐seq data from NCBI BioProject PRJNA439019 (SRR6924534–SRR6924549).

Raw sequencing reads were processed as previously described to generate count matrices. Genes with fewer than five counts in at least 12 samples were excluded, yielding a filtered dataset of 13 284 genes for network analysis. Expression values were normalised using the variance‐stabilising transformation (VST) from DESeq2 (Anders and Huber 2012). The co‐expression network was constructed using the WGCNA package (v1.70‐3) in R (Langfelder and Horvath 2008), applying the following parameters: unsigned network type, soft‐thresholding power of 10 (determined by scale‐free topology criteria), maximum block size of 14 000, and a mergeCutHeight of 0.25. Gene modules were identified using the dynamic tree‐cutting algorithm with default settings. To quantify co‐expression relationships, an adjacency matrix was computed using the selected soft power, followed by generation of a topological overlap matrix (TOM) via the TOMsimilarity function with the ‘unsigned’ option. For visualisation and downstream analysis, gene pairs with TOM values greater than 0.1 were retained, representing robust co‐expression links.

## Author Contributions

Á.P., A.P.‐G. and L.J.‐C. designed and planned the experiments. A.L.‐L. and L.J.‐C. performed experiments and data analysis. L.J.‐C. and Á.P. wrote the manuscripts. A.P.‐G. and D.F.‐O. revised the manuscript. Á.P. and A.P.‐G. supervised the study. All authors read and approved of its content.

## Funding

This work was supported by AYUDAS A LA I+D+i EN EL ÁMBITO DEL PLAN ANDALUZ DE INVESTIGACIÓN, DESARROLLO E INNOVACIÓN (PAIDI 2020) (PY20_00048) and MICIU/AEI/10.13039/501100011033 and ERDF/EU (PID2022‐136240OB‐C21). Funding for open access charge: Universidad de Málaga / CBUA.

## Conflicts of Interest

The authors declare no conflicts of interest.

## Supporting information


**Appendix S1:** pbi70526‐sup‐0001‐AppendixS1.zip.

## Data Availability

The data that support the findings of this study are openly available in LUCID at https://github.com/Brunxi/LUCID.
